# Perceptions Towards HCV Treatment with Direct Acting Antivirals (DAAs): A Qualitative Analysis with Persons with HIV/HCV Co-infection Who Delay or Refuse Treatment

**DOI:** 10.1007/s10461-022-03749-8

**Published:** 2022-07-01

**Authors:** Sarah Brothers, Elizabeth DiDomizio, Lisa Nichols, Ralph Brooks, Merceditas Villanueva

**Affiliations:** 1 Department of Sociology, Pennsylvania State University, 316 Oswald Tower, University Park, PA 16802 USA; 2 HIV/AIDS Program, Yale University School of Medicine, Section of Infectious Diseases, New Haven, CT USA

**Keywords:** HIV/HCV co-infection, Hepatitis C treatment, Direct-acting antiviral treatment, Comorbidity, Qualitative interview

## Abstract

In the United States, approximately 25% of people with HIV (PWH) are co-infected with hepatitis C (HCV). Since 2014, highly effective and well-tolerated direct-acting antivirals (DAAs) have revolutionized HCV treatment. Uptake of DAAs by people with HIV/HCV co-infection has improved but remains suboptimal due to system, provider, and patient-level barriers. To explore patient-level issues by better understanding their attitudes towards DAA treatment, we conducted qualitative interviews with 21 persons with HIV/HCV co-infection who did not consent to DAA treatment or delayed treatment for at least 1 year after diagnosis. We found PWH perceived DAA treatment barriers and facilitators on multiple levels of the social-ecological environment: the individual (HCV disease and treatment literacy), interpersonal (peer influence), institutional (media and healthcare provider relationship), and structural levels (treatment cost and adherence support). Recommendations to improve DAA treatment uptake include HCV-treatment adherence support, HCV disease and treatment literacy training (particularly for substance use and DAA treatment interactions), and encouraging PWH who have successfully completed DAA treatment to speak with their peers.

**Supplementary Information:**

The online version contains supplementary material available at 10.1007/s10461-022-03749-8.

## Introduction

In the United States, approximately 25% of people with HIV (PWH) are co-infected with hepatitis C (HCV) [1, 2], which is the leading cause of liver-related morbidity and mortality in the country [3–5]. HIV/HCV co-infection accelerates liver disease progression, and co-infected people are disproportionately affected by liver-related mortality and elevated mortality in general [6–10]. HCV infection is increasing due to the opioid epidemic, and it is estimated that 62- 80% of people who inject drugs (PWID) living with HIV are co-infected with HCV [11–13].

In 2014, the introduction of direct-acting antiretroviral (DAA) treatment in the United States revolutionized HCV treatment. DAA treatment is highly effective (> 95% cure rates), well-tolerated, and can be administered for short courses (8–12 weeks for current pan-genotypic regimens) [14, 15]. Unlike the previous interferon-based treatment, DAA treatment is just as effective for persons with HIV/HCV co-infection as it is for people with HCV mono-infection [16–19]. Due to the availability and efficacy of DAA treatment, the World Health Organization (WHO) launched an initiative to eliminate HCV as a global public health threat by 2030 [20]. The CDC Division of Viral Hepatitis 2025 Strategic Plan aims to increase HCV cure to > 85% by 2030 [21].

However, despite DAA treatment’s availability, ease, and efficacy, uptake is insufficient to meet HCV elimination goals [22]. Most people diagnosed with HCV are still untreated [6, 23–25]. Targeting the smaller group of PWH with HCV for micro-elimination efforts has been proposed, given the higher morbidity and mortality in this group [11, 26–28]. While various studies show that implementation efforts to expand HCV treatment in persons with HIV/HCV co-infection result in improved cure rates [29–35], there remains a treatment gap that poses an ongoing barrier to micro-elimination efforts. Thus, meeting HCV micro-elimination goals among persons with HIV/HCV co-infection requires analyzing treatment barriers and facilitators for PWH who delay or do not consent to DAA treatment.

With few exceptions [36], previous research on DAA treatment barriers and facilitators has primarily focused on people with HCV mono-infection where multiple issues influence DAA treatment uptake, including alcohol and drug use, socio-economic variables, homelessness, comorbidities, social support, knowledge of HCV symptoms and treatment, provider relationships, and residual fear of treatment side effects from the pre-DAA interferon era of treatment [6, 14, 36–39]. Among co-infected persons, barriers to establishing HCV care include mental health disease, ongoing drug use, being non-white, CD4 < 200, and detectable HIV viral load [39]. While demographic analysis can shed light on the ongoing multifactorial treatment gap, qualitative research is critically important for gaining an in-depth understanding of previously unidentified processes that influence patient-level perceptions of DAA treatment by PWH who delay or refuse treatment. Such understanding is essential for developing interventions to improve treatment uptake among PWH with HCV and may contribute to interventions for increasing HCV treatment uptake in general.

We use a modified social-ecological model [40, 41] to examine the multiple interconnected themes on the individual, interpersonal, institutional, and structural levels that serve as barriers or facilitators to DAA treatment for persons with HIV/HCV co-infection who delay DAA treatment for at least 1 year after diagnosis or do not consent to DAA treatment.

## Methods

The methodology chosen for this study is Charmaz’s constructivist grounded theory, which emphasizes an inductive and iterative approach that allows themes to emerge from participants’ lived experience and helps capture theoretical insights from the data [42]. Semi-structured qualitative interviews were selected over focus groups to help ensure participant confidentiality [43] and gain an in-depth understanding of processes that influence patient-level HCV treatment perceptions. This paper follows the SRQR guidelines for reporting qualitative research [44].

### Patient Recruitment

From April 2020 to February 2021, the first author, who has extensive training and experience conducting semi-structured interviews and working with vulnerable populations including PWH, PWID, and people experiencing homelessness, conducted in-depth interviews with 21 people who had a confirmed HIV and HCV diagnosis and who delayed treatment for HCV for at least 1 year after HCV diagnosis or had not consented to treatment at the time of the interview. Patients were recruited from seven HIV primary care clinics located in urban areas in Connecticut (CT); they serve a PWH population comprised of racial and ethnic minorities (African American and Latinx) with major HIV transmission risk factors being men having sex with men (MSM) and historically PWID. Clinics were university-based or Federally Qualified Health Centers. All clinics receive Federal Ryan White funds and have a multi-disciplinary team approach with access to medical case managers and mental health and substance use providers in addition to Infectious Disease (ID)-trained HIV providers. Designated on-site ID providers with special training in HCV management provided HCV treatment. In some cases, the patient’s HIV provider (responsible for HIV antiretroviral treatment (ART) and primary care management) was also the HCV prescriber; in other cases, the HIV provider referred the patient to the on-site HCV provider through a standardized algorithm [39] where all treatment plans were available in the electronic medical record (EMR).

This study was part of a HRSA Special Project of National Significance (SPNS) initiative Curing Hepatitis C among People of Color Living with HIV. The goal was to assess and promote efforts on a clinic and statewide level to treat HCV among persons with HIV/HCV co-infection. All patients with HIV/HCV co-infection in CT were identified by a surveillance-based algorithm and were entered into a centralized database from which HCV treatment status was tracked based on laboratory data routinely collected by the CT Department of Health (DPH); all patients in the database were given a unique DPH code. Patients in the centralized database were filtered by the participating clinic to generate clinic-based lists.

Eligible patients were 18 years of age or older, able to speak English, had delayed HCV treatment for at least 1 year after diagnosis, and at the time of the interview were either untreated for HCV, had fully completed treatment for HCV in the past year, or were currently in treatment. We included patients who had recently entered treatment after previously refusing treatment because they could provide valuable information about the processes surrounding their delay of DAA treatment and those that facilitated their entry into treatment.

To recruit patients, in March 2020, the fourth author collected de-identified clinical data from each of the participating clinics housed in the centralized database; this included demographics and the most recent HIV and HCV PCR results. The list of untreated patients (chronically infected as defined by PCR positivity) was then sent to each participating clinic. In July 2020, clinics further identified patients treated in the previous year based on the treatment initiation date. Patients in the centralized clinic database were given a DPH code, and patient identifiers were available only to clinic staff. Clinic staff contacted potentially eligible patients, and those who were interested were referred to the first author to learn more about the study. Phone calls were initiated by clinic staff familiar with the patient who could confirm their identity. The first author screened all participants by phone to verify eligibility. Eligibility questions included: HIV and HCV status, date of diagnoses, HIV and HCV treatment status, if they had ever received treated for HCV, and if so, the date of treatment. If eligible, a phone interview was scheduled. Prior to each interview, the interviewer clarified to participants that she was independent of their clinical treatment programs, and individual information was confidential and not shared with their clinics.

### Interviews

The interviews were an extended guided conversation in which participants were encouraged to introduce topics, ask questions, expand on their answers, and share their perspectives on their personal experiences [45]. This method was chosen to ensure that core domains were discussed, and unexpected themes could emerge, consistent with the grounded theory approach [42]. Interviews loosely followed an interview guide informed by sensitizing concepts drawn from previous literature on barriers and facilitators to HCV treatment (i.e., [31, 32, 46–48]). It included the following domains, which the interviewer used to evoke responses detailing attitudes towards DAA treatment: participant characteristics and demographics, HIV and HCV diagnosis and treatment history, knowledge and perceptions of HCV disease and treatment, provider relationships, social support, religion, comorbidities, health and wellbeing, stigma, history of substance use, housing and employment, food insecurity, and transportation issues. The guide was developed by the first, third, and last author and was refined multiple times.

The semi-structured in-depth interviews were 40–70 min in length, open-ended in nature, and audio-recorded. Due to COVID-19, all interviews were conducted via telephone without video while participants were in privacy in their homes. Participants were read the consent form and gave informed consent before engaging in the interview. Participants received a $40 gift card in compensation for their participation. The study received ethical approval from Yale University School of Medicine.

### Data Analysis

Qualitative analysis was conducted using constructivist grounded theory [42]. Thus, we iteratively collected and analyzed the data. After each interview, the first author wrote analytic memos to identify categories emerging from the data and additional memos noting how themes recurred throughout the interviews.

The team sought to conduct approximately 20 qualitative semi-structured interviews. Over the course of data collection, emergent themes became redundant, suggesting that thematic saturation was reached, all major themes were identified, and an adequate sample size had been reached [49]. At this point, we ceased recruitment [50].

The interviews were professionally transcribed verbatim. The first author checked the transcriptions for accuracy against the audio files and read all interviews multiple times. Interview transcripts were entered into ATLAS.ti software for data management and analysis. The first author analyzed the transcripts throughout data collection by conducting open coding and writing analytic memos on emergent themes and patterns. The coding framework was refined through further readings of the transcripts and coding reports and writing additional analytic memos to refine the codes.

After data collection was complete, the second author (also trained in qualitative methods) independently coded eight transcripts. The first and second authors then developed a codebook describing the key themes and conducted focused coding to refine the list of codes further, modify the codebook, and develop the themes. Next, they generated coding reports that collected quotes for each theme. They iteratively analyzed the coding reports and the full interviews for the quotes’ context to refine the code book and generate the specific coding for the final themes in this analysis. Any coding discrepancies were discussed by reviewing the text coded differently until the discrepancies were fully resolved by consensus. The entire team met several times throughout the process to discuss emergent themes, resolve any differences in interpretations, address uncertainties, develop concepts and relationships between the open codes, and discuss coding categories. Categories were not predetermined. Instead, they emerged inductively through data collection and analysis. Once the researchers reached a consensus on a revised codebook, the first and second authors used the final codebook to code all transcripts. When coding was complete, the codes were organized into common themes, and the themes were then organized into categories that reflected DAA treatment barriers and facilitators.

Using Charmaz’s approach, emergent categories were compared with the literature during the analytic process to strengthen and clarify the analysis [42, 51]. In Charmaz’s approach to grounded theory, theoretical frameworks must fit the data and be justified by their capacity to enrich the analysis. It became clear that DAA treatment barriers and facilitators were determined on multiple levels and we chose the social-ecological model as an optimal theoretical framework for the data. Drawing on work that theorizes that multiple levels influence HIV-related behaviors [41], the team used a modified social-ecological model as the framework for the results. This model fosters the examination of how multiple themes in the structural, institutional, interpersonal, and individual contexts influence health. The individual-level includes individual beliefs and behaviors. The interpersonal level contains relationships with friends, romantic partners, and family. The institutional level includes local institutions such as medical clinics. The structural level includes socio-economic forces such as social policies and bureaucratic systems. Quotations are identified by gender identity, race/ethnicity, and DAA treatment status. Age has been omitted to ensure confidentiality.

## Results

### Participant Characteristics

Overall, 21 patients were recruited: 9 were untreated for HCV, and 12 entered DAA treatment in the past year (Table 1). Participants ranged in age from 39 to 70 (mean 59 years). Ten participants identified as Black, five as Puerto Rican, five as white, and one as Native American. Eleven of the participants identified as women and ten participants as men. Twenty participants identified as heterosexual; one participant identified as LGBTQ. Fifteen participants had a self-reported history of injection drug use, and all participants had a history of substance use disorder (SUD). Median years since HCV diagnosis was 15 years (range 2 to 36 years) and was similar between those untreated and those recently treated. Median years since HIV diagnosis was 25 years. At the time of the interview, 20 participants received antiretroviral therapy (ART) as prescribed by their HIV clinic provider. Of the 12 participants who entered DAA treatment in the past year, eight self-reported they had completed treatment, and four were still in treatment at the time of the interview (data not shown).

**Table 1:** Participant characteristics (N = 21)

	DAA treatment last 12 months n = 12	Untreated for HCV* n = 9	Total N = 21
Gender	n	n	n
Male	7	3	10
Female	5	6	11

Race/Ethnicity	n	n	n
Black/African American	7	3	10
Puerto Rican	1	4	5
White	3	2	5
Native American	1	0	1

Age	Mean	Mean	Mean
Range: 39 to 70 years	61	57	59
LGBTQ?	n	n	n
Yes	0	1	1
No	12	8	20
History of injection drug use?	n	n	n
Yes	11	4	15
No	1	5	6

Years since diagnosis	Median	Median	Median
HIV	26	23	25
HCV	14	16	15
Ever received interferon-based treatment?	n	n	n
Yes	1	0	1
No	11	9	20
Currently on antiretroviral HIV treatment?	n	n	n
Yes	12	8	20
No	0	1	1

*At time of interview

### Study Themes

**Fig. 1:**
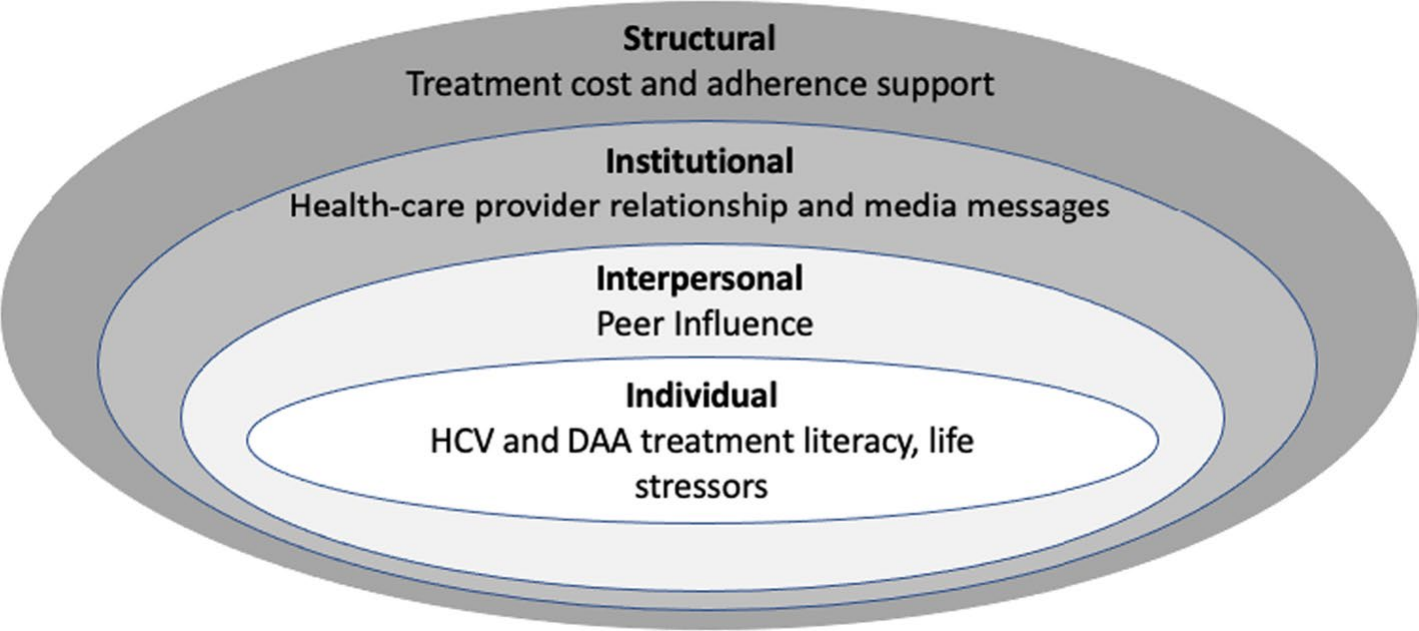
Modified social-ecological model for themes that influence DAA treatment decisions by people with HIV/HCV co-infection who delay or refuse treatment

We identified multiple themes across four levels of the social-ecological model that positively or negatively influenced participants’ DAA treatment perspectives. On the individual level, themes included HCV disease and treatment literacy, fear of side effects, and complex life stressors. On the interpersonal level, themes included information received from peers. On the institutional level, healthcare provider relationships and media messages were key. On the structural level, treatment cost and adherence support influenced treatment perspectives (Fig. 1).

There were no differences between participants in themes expressed for negative influences on DAA treatment perspectives by demographics, time since HIV or HCV diagnosis, or between treated or untreated participants. All participants had refused DAA treatment at least once in their medical history. Participants who later changed their perspective and entered DAA treatment reported that their treatment perspectives were positively influenced by receiving information about DAA treatment from peers, changing their provider, developing a trusting relationship with a provider, or receiving adherence support.

### Individual Level Themes

This section first describes how, on the individual level, limited literacy of HCV disease and DAA treatment negatively influenced treatment perspectives. In particular, fears of side effects and beliefs that active substance use decreases DAA treatment efficacy and makes HCV infection inevitable were barriers to treatment. Next, this section discusses how complex life stressors were DAA treatment barriers.

#### HCV Disease and Treatment Literacy

Knowledge deficits of HCV disease symptoms, severity, and DAA treatment side effects and contraindications were treatment barriers. Some participants who refused treatment were unfamiliar with HCV symptoms and believed them to be mild. One man said of HCV:

I don't know what the symptoms are. Sluggish? I'm sometimes sluggish. (Participant #4, Black Man, untreated)

Participants described perceiving they did not need HCV treatment because they were not experiencing symptoms such as weight loss, stomach pains, fevers, or breathing difficulties. A few stated they thought of HCV as an inevitable but relatively benign side effect of HIV infection.

A quarter of participants described fears of side effects as a barrier to treatment. Instead of perceiving DAA treatment as beneficial to their long-term health, some participants refused treatment because they were afraid DAA medication would make them feel worse, illustrating how lack of knowledge can influence motivation to enter treatment. One woman who was diagnosed with HCV over 20 years ago explained she was not interested in DAA treatment because she did not feel she was experiencing symptoms and thought treatment could adversely affect her health:

I'm not losing weight. I'm not having stomachache. I'm not having fevers. I never have any pneumonia in my life. At this time, I don't feel I should I take it. Why would I wanna start a medication that could start making me feel like I don't wanna feel? (Participant #8, Puerto Rican Woman, untreated)

Participants who previously experienced side effects from ART or other medications described those experiences as barriers to DAA treatment. One man said he left ART and refused DAA treatment for years because of a negative experience with medication side effects:

The medication was not improving my health. If anything, I began deteriorating. So, once I started deteriorating, I was, like, "Well, look. Hold up. So, you turned around and you put me on the medicine, and this is happening? No, I'm going to go it on my own. You and your medication, keep that away from me." I'm real funny about medications, there's some things I won't take. Because I'm afraid of the side effects and I think you're trying to kill me. (Participant #7, Black Man, completed treatment)

Some participants perceived their health as precarious and feared that an additional treatment could adversely effect their overall health. One woman said:

[My provider] tries to bring it up on almost every occasion. But I have this intuition feeling that just won't let me do it. He tries valiantly. But something is not gonna go right. I'm gonna get very, very sick. It might back a flare up of hepatitis B on top of everything. I am not gonna be able to function, I'll become weak and not be able to get out of bed and that ain't happening. (Participant #3, White Woman, untreated)

Over a third of participants believed active alcohol and substance use were barriers to DAA treatment. Some thought they had to be abstinent before, during, and after treatment. For example, one participant refused treatment because she believed she had to stop drinking alcohol before DAA treatment: “But at time I couldn't stop drinking, didn't want to stop drinking, so I put it off.” (Participant #2, Black Woman, untreated).

Participants described perceiving that side effects would be more severe, or treatment efficacy reduced if they consumed alcohol or illicit drugs during treatment. Some believed HCV would return if they were not abstinent after treatment. However, participants did not perceive that drug or alcohol use would decrease their DAA adherence. They reported adhering to other medications, including ART, while actively consuming alcohol and illicit substances.

#### Complex Stressors

The complex life stressors faced by some participants were barriers to DAA treatment. Some wanted to address other health issues before starting DAA treatment, particularly their HIV viral load. One woman said:

I didn't even think about it yet. I'm trying to get my HIV stuff under control. I guess I should [get treated for HCV], but I just need to get one thing under control at a time. Then I'll do the hepatitis thing. (Participant #1, Black Woman, untreated)

Another participant delayed treatment because she felt too overwhelmed by her multiple health needs to initiate DAA treatment:

There were times when I wouldn't follow through with the referrals because I had so much going on, and it's just too much on my head sometimes. I have HPV; I have this, I have that, so I gotta take care of everything, I have GI issues, I have a lot of things going on. (Participant #21, White Woman, completed treatment)

Several participants delayed DAA treatment because they believed they were already being treated for too many issues and did not want to add additional medications to their regimen. One woman said she initially delayed DAA treatment because:

I already take enough medication. I didn't want another pill. (Participant #12, Black Woman, completed treatment)

Participants also reported refusing DAA treatment because they wanted to improve their health before beginning treatment by improving their diet, losing weight, or stopping smoking. Some discussed their desire to complete outstanding tasks such as changing residences before starting treatment because they believed side effects would debilitate them during treatment.

### Interpersonal Level Themes

This section begins by discussing interpersonal level barriers to treatment. First, information received from peers, including residual information about interferon’s side effects and limited efficacy, served as barriers to treatment. Next discussed is peer perceptions that HCV is benign. Then, peer perceptions that abstinence is required for DAA treatment. Finally, this section examines how peers facilitate DAA treatment. Several participants who delayed treatment described being motivated to enter treatment by peers’ first-hand information about the ease and efficacy of DAA treatment.

#### Peer Perceptions of HCV Disease and Treatment

Residual information received from peers about negative experiences with interferon-based treatment made some participants reluctant to start DAA treatment. Peer information sometimes outweighed information received from providers. For instance, one participant delayed DAA treatment because the information he received from his friends and acquaintances about side effects and treatment efficacy contradicted the information he received from providers:

By hearing all the side effects, I was just dissuaded against doing it. I was getting that information on the ground- on the street level, and my other information was coming from other providers. I did my research and found out how successful it is, but I had to do my research first. (Participant #10, Black Man, completed treatment)

In addition, peer perceptions that HCV was a benign disease were a treatment barrier. Some participants described how their families were engaged in their HIV treatment, including ensuring they attend appointments and maintain adherence, but did not encourage them to enter DAA treatment or consider it a priority. One woman said,

We don't focus on it. And really, I haven't talked about it in years with my children. (Participant #3, White Woman, untreated)

Secondhand knowledge of peers’ disease progression experiences was another barrier. Some participants said they refused DAA treatment because they perceived their untreated peers were healthy. One woman said:

The hep was not priority for me. I didn't realize what it could do, and I just thought people lived with it. I knew people that had it and they were 70. So, it, to me, it wasn't a big deal. (Participant #17, Native American Woman, completed treatment)

A notable treatment barrier was information received from peers that alcohol and substance use during treatment caused severe side effects. One woman said:

I heard people in my program and out in the street talking, whenever you take the pill for the hepatitis, you can't drink or nothing. You can't do drugs. If you drink or do drugs, you gonna get sick, a lot of people told me. (Participant #11, Black Woman, completed treatment)

Several participants who actively consumed alcohol and illicit substances described refusing treatment because peers said they needed to stay abstinent after treatment or HCV would return. Some seemed to interpret peer information on substance use-related HCV reinfection risk to mean that HCV infection was linked to substance use in general rather than specific practices such as sharing syringes. One man who injected opioids said he refused DAA treatment because peers told him HCV would recur if he did not stay abstinent. He said:

The only thing I know is what my friends have been telling me. ... if I stay clean, they will cure me. It's only three months, and in three months, they take your blood, and they check and hey, you don't got it. But if you go back and fool around again, you get it right back, the hepatitis C come right back. (Participant #15, Puerto Rican Man, untreated)

#### Peer Experiences with DAA Treatment

A minority of participants said peers who successfully completed DAA treatment facilitated their decision to start treatment. One participant, who previously refused treatment because she felt she was taking too many medications to treat comorbidities, planned to start treatment next month. She said:

My friend told me that I should go do it because it ain't that hard of a treatment. She did it last year. And she said it wasn't that bad at all. She said nothing happened with her. (Participant #1, Black woman, untreated)

Another participant entered DAA treatment after learning that his nephew had completed treatment. Then he, in turn, influenced his ex-wife’s decision to start treatment:

She asked me, and I said, "Yeah, well, I don't have it anymore." I kept telling her that. I said, "It's easy." Because she don't really like taking pills. I said, "All you got to do is take one pill a day, that's it. It won't be no big horse pill. It's just one pill you take every day, and after eight or 12 weeks, the worst is gone.” (Participant #5, Black Man, completed treatment)

### Institutional Level Themes

This section begins by discussing institutional-level barriers to treatment. First, how media advertisements are perceived as barriers to treatment. Next, it describes how weak and transitory relationships with healthcare providers and communication issues between providers and patients about interactions between alcohol and substance use and DAA treatment negatively influence treatment decisions. Finally, this section examines how stable, persistent, and supportive relationships with providers facilitated DAA treatment.

#### Media Messaging

Some participants delayed DAA treatment because they did not trust the information conveyed by media advertisements. They said overly positive media messages made them skeptical about DAA treatment because they felt the messages did not represent their experiences or present a balanced, honest perspective:

I kept seeing commercials on TV.‬ “Oh, you can be at the beach. Oh, you'll be feeling chipper in a day.‬” That's the shit that I was seeing. I was, like, yeah, right. They're not telling you, “Oh you can be suffering from this. You can suffer that.” You telling me that I can be out horseback riding or mountain climbing and at the beach.‬ (Participant #7, Black Man, completed treatment)

Some felt that they received conflicting messages from media and other sources of information. This disagreement caused some to refuse DAA treatment because they did not know which source of information to trust. One man delayed both HIV and DAA treatment because he felt confused and scared by conflicting media messages:

I was scared. It was so many different... the TV and the news and the radio, “This medication is good. That medication is killing people.” (Participant #14, Puerto Rican Man, untreated)

#### Weak and Transitory Provider Relationships

Some participants described refusing DAA treatment because their healthcare providers changed frequently; thus, they did not have a consistent relationship with any provider, including an HIV provider. They described feeling that they invested time disclosing to one provider but by the time they felt their provider was familiar with their case, they were assigned to a new provider, so they never considered additional treatments such as DAA treatment.

Some said they started discussing DAA treatment with a provider and considered entering treatment, but they did not resume the conversations when their provider changed. Participants also described feeling uncomfortable sharing personal information with multiple providers and worried about privacy violations, especially through disclosing potentially stigmatizing information. For instance, one participant described how he suffered anxiety over disclosure and interrupted care because he lacked a consistent provider. He said one provider suggested DAA treatment, but then his provider changed, and treatment was not mentioned again:

I never saw that doctor again. I don’t know what happened. I just know that he talked to me about it. I had a problem in that clinic, every month that I used to come to the clinic, every month was a different doctor.‬ It was like the whole New Haven knew that I was HIV. So, that was bothering me. (Participant #14, Puerto Rican Man, untreated)

Participants who lacked a trusting relationship with their regular provider perceived their poor relationship as a treatment barrier. Some described refusing DAA treatment because they thought their providers lacked concern for their overall wellbeing, were not honest with them, or were dismissive of their experiences with side effects. One participant said she refused DAA treatment and was out of HIV care for years because she felt her provider did not properly address her HIV medication side effects:

He put me on a medicine that was making me sick, and I said, “Can’t you give me something else?” And all he said is, “You’re resourceful. You’ll find a way.” And that just kind of pissed me off, so I stopped going to him. I was out of care for a while. (Participant #21, White Woman, completed treatment)

#### Provider Communications About Alcohol and Substance Use

Communication issues between patients and providers about active alcohol and substance use during DAA treatment were a common barrier. Some participants said they refused DAA treatment because their provider told them they needed to be abstinent prior to treatment. One woman delayed DAA treatment because her provider said she needed to stop drinking alcohol for the treatment duration. She stopped drinking alcohol for 2 months with great difficulty: “cause I've been drinking all my life. So, I had to go them two whole months without drinking, but I got used to it” (Participant #11, Black Woman, completed treatment).

Participants described interpreting provider communications advocating sobriety because of HCV reinfection risks from continued alcohol and substance use to mean that HCV is a chronic condition that could recur if they were not abstinent after treatment. One man said his doctor told him:

That now I was fine and that I don't have Hep C anymore, but to make sure that I don’t start drinking alcohol and stuff because it could return. (Participant #6, Black Man, completed treatment)

#### Stable Supportive Provider Relationships

Three-quarters of participants who initiated DAA treatment after delaying did so after establishing a stable and supportive relationship with a provider who they perceived approached them as a whole person rather than a patient. Providers fostered these relationships by exchanging personal non-medical information about families and social interests. These conversations helped build a trusting relationship where the patient was motivated to strongly consider the provider’s treatment recommendations.

Several participants entered treatment because their provider carefully explained HCV disease and DAA treatment options over multiple visits, emphasized they had a curable condition, and allowed them time to make treatment decisions. For instance, one participant, after refusing treatment for years, entered DAA treatment because he was assigned a provider who suggested DAA treatment at every visit, carefully explained how HCV disease would progress, and let him decide at his own pace:

The one I got now is the one that really convinced me to do it. She told me the long-term effect that it could have if I don't. And so, I just stopped and thought about it. It took a little while. She suggested it the first time, I told her I would think about it, and I came back to see her maybe two more times. She said, "Have you made up your mind?" And I said, "No, but I'm going to do it anyway." It wasn't a whole lot of talking about it, but she was just telling me the good side about it and her side about it. And, because she's a straight-up lady I said, “Oh okay, fine.” (Participant #5, Black Man, completed treatment)

Participants described deciding to enter DAA treatment after being assigned to a new provider who addressed their concerns about side effects. Such providers carefully explained potential side effects based on their medical profile and assured them they would stop DAA treatment if they experienced side effects. One participant resumed ART and then entered DAA treatment because his new provider told him that she would immediately stop treatment if he experienced adverse side effects. He said,

I was reluctant to do it because I was afraid of any effect. She said, “Well, look, any type of mishap, we're going to pull back. We're going to take you off of it right away.” ..... if it wasn't for [my doctor], I wouldn't have never did it. (Participant #7, Black Man, completed treatment)

Participants who perceived that a provider had protected them from side effects from other medications said the experience facilitated their entry into DAA treatment. They described following DAA treatment recommendations from providers who watched for interactions between their HIV medications and other medications, researched potential side effects based on their specific medical history, and carefully explained possible side effects:

I trust her opinion and her decision. She makes sure that my mental health medicine don't interfere with my HIV medicine. She said I could have a reaction if I take the wrong ones with the wrong one. (Participant #13, Puerto Rican Man, completed treatment)

One participant who delayed DAA treatment because she experienced negative side effects from interferon entered treatment because her provider assured her DAA side effects would be minor, he would adjust treatment if she experienced ill effects, and he would research possible interactions with her medications:

He assured me that they weren't showing those symptoms with people, so we'll give it a shot. If I went to them and said, "This is bothering me." they probably would have adjusted it and they would have gave me something different... My doctor is very good at saying to me, "No, I'm not sure if this will interact correctly, but I'm going to do some research and then we'll talk about it the next time.” (Participant #17, Native American Woman, completed treatment)

Participants also reported that a strong provider relationship could overcome individual level and interpersonal level perceptions of DAA treatment barriers, such as fears of side effects and discouraging information received from peers. For instance, participants with multiple comorbidities who delayed treatment because they did not want to take additional medications described how providers overcame their reluctance. They framed DAA treatment as a relatively speedy treatment that would eliminate one of their many health issues. Additionally, some participants whose decision to enter treatment was facilitated by developing a solid relationship with a new healthcare provider were concurrently motivated on the interpersonal level through information received from peers who completed DAA treatment.

### Structural Level Themes

This section first describes how financial considerations were barriers to treatment on the structural level. Next, it examines how support for medication adherence, such as directly observed therapy (DOT), facilitated DAA treatment.

#### Cost of DAA Treatment

A minority of participants who delayed or refused DAA treatment said they did so because of the high cost of treatment. A few participants delayed treatment until they received health insurance because of personal expense. One man said:

I didn't have health insurance. The only thing we had was the state insurance, and they wouldn't pay for it, and it's like $80,000 for that treatment. (Participant #18, White Man, completed treatment)

However, the cost was a barrier even for many participants who would incur no personal expense for treatment. Several participants believed insurance would only pay for treatment once, so they delayed treatment until they thought they could complete it:

I just want to be where I'm stable and going to commit to it because insurance will only pay for it once. So, you mess it up, you don't get it again. The Hep C could kill me before the HIV would. I'm just procrastinating. I'm afraid that if I do it, and I don't do it right... I'm terrified of that. (Participant #20, White Woman, untreated)

For some, the cost of treatment exacerbated their low self-esteem to create treatment barriers. They described refusing DAA treatment because they did not feel they deserved such an expensive treatment until they improved themselves by losing weight, quitting smoking, or ceasing alcohol and substance use. One man described refusing treatment. He felt he did not deserve it because he consumes illicit drugs and has no family to care for:

Each pill cost $1,100. If you know you're gonna keep fucking around, why you wasting all that money, man? You should leave that to somebody who really want to stay clean or somebody who got a baby daughter, baby boy, a beautiful girl, a companion, whatever. But if you know you're gonna fuck around, yo, keep fucking around, die. (Participant #15, Puerto Rican Man, untreated)

#### Adherence Support

A minority of participants described adherence concerns as a barrier. Some participants who delayed treatment described entering treatment after they received adherence support, particularly directly observed therapy (DOT), in which a health care professional observes the patient take their medication dose. Several participants who received DOT for ART said it gave them the support they felt they needed for DAA treatment. For instance, one participant, who stopped taking her HIV medication 6 months prior to the interview when her DOT program stopped, said she plans to start DAA treatment after her DOT program resumes:

I used to have a nurse that come and distribute them, but I don't have that no more, so I don't remember to take my meds. I stopped taking them ‘cause there was nobody looking over my shoulder. And now I'm about to start taking them back when they send a nurse to distribute them to me. (Participant #1, Black Woman, untreated)

Some participants devised other methods, including self-devised daily alarms, to support their adherence. One participant programmed her Amazon Alexa virtual assistance AI technology to remind her to take her HCV medication. Another participant, who delayed DAA treatment because she did not think she could maintain adherence, started treatment after she received electronic DOT for other medications in the form of an automatic pill dispenser with timed alerts:

[My doctor] started telling me my liver was hardening and stuff. She was asking me about things that were going on, it kind of scared me. I don't need my liver to break too, I got enough problems. At first, I wasn't too sure about it [DAA treatment] because sometimes I'm not med adherent but I have a med box now so I'm good with everything. It's on timers. It goes off at 9:00 in the morning, 12:00 in the afternoon, 4:00 in the afternoon, and then 9:00 at night. (Participant #21, White Woman, completed treatment)

## Discussion

To our knowledge, this is the first study to explore perceptions towards DAA treatment by persons with HIV/HCV co-infection who delay or do not consent to treatment. This qualitative study provides important insights into barriers and facilitators to HCV treatment during the DAA era for this understudied and high-risk population.

Multiple interconnected themes on the individual, interpersonal, institutional, and structural levels influenced their uptake of DAA treatment. These included: (1) Individual level: DAA treatment and HCV disease literacy; (2) Interpersonal level: information from peers; (3) Institutional level: provider relationship and media; (4) Structural level: treatment cost and adherence support (Table 2).

**Table 2:** Barriers and Facilitators to DAA treatment

Barriers	Facilitators
• Misconceptions about DAA treatment abstinence requirements	• Increased provider literacy that substance use is not a contraindication for DAA treatment • Increased patient literacy on DAA treatment safety and efficacy during active substance use
• Not prioritizing HCV treatment	• Provider discussions about importance of HCV treatment
• Fear of side effects and interactions	• Provider discussions about DAA side effects and interactions
• Peer-received information on interferon treatment	• Peer-received information on DAA treatment • Provider-received information on differences between Interferon treatment and DAA treatment
• Transitory provider relationships	• Stable and trustworthy provider relationships with longitudinal discussions about benefits and side effects of DAA treatment
• Distrust of media messaging	• Provider discussions of DAA side effects and treatment efficacy
• Treatment cost	• Provider discussion that patient is deserving of DAA treatment
• Adherence support needs	• Implement adherence support such as directly observed therapy (DOT), automated reminders

On the individual level, many participants delayed DAA treatment because of fear of side effects. Residual information about interferon-based treatment side effects was a common barrier, as prior studies have found [14, 30]. Patients may need explicit information on how DAA treatment differs from interferon-based treatment, including its mechanisms, side effects, length of treatment, and cure rate. Moreover, community messaging such as testimonies from HCV treatment advocates, fact sheets, pamphlets, talks, and webinars should fully address the differences between interferon-based treatment and DAA-based treatment. For example, our group has created a patient app and website that helps patients navigate the HCV treatment landscape through a series of modules.

Our study also finds that many participants’ fears of side effects arose from their experiences with adverse side effects and drug interactions from various medications throughout their medical history, including ART. Providers should consider obtaining a complete history of experiences with side effects to find the roots of their distrust. In addition, they should fully describe the symptoms and potential side-effects of DAA treatment so patients understand what may happen. They should also reassure patients that they are unlikely to experience severe side effects during DAA treatment, but they can stop treatment if they do.

Perceptions that active alcohol and substance use is a contraindication for DAA treatment were barriers on the individual, interpersonal, and institutional levels. Studies have found that people who actively use alcohol and illicit substances have lower treatment uptake rates [14, 52, 53]. Misinformation about DAA treatment abstinence requirements may be a barrier. Patients may need more detailed guidance on how active alcohol and substance use does not decrease DAA treatment efficacy or increase side effects. In addition, some participants stated that their providers required abstinence for DAA treatment. Although active alcohol and substance use are not contraindications to DAA treatment and people who actively use drugs or alcohol have sustained virological response (SVR) rates comparable to those who do not [54–56], some providers still require abstinence to initiate DAA treatment [57, 58]. Providers may need more information that patients’ active use of alcohol and other substances are not contraindications to DAA treatment and training on patients’ active use of alcohol and other substances during DAA treatment to change their attitudes and perspectives. In addition, since HIV/HCV co-infection is particularly high among PWID [11, 12], providers may need information on shaping DAA treatment for people who currently or formerly injected drugs. Moreover, because of the potential HCV reinfection risk associated with relapse, particularly of injection drug use, people with ongoing substance use disorders require more information on how future substance or alcohol use may impact their risk of HCV reinfection and should be given resources to reduce this risk.

Participants’ lack of knowledge about HCV symptoms and their perceptions that HCV treatment was not a priority were barriers to treatment in this study, similar to research on PWID with HCV mono-infection [32]. This study adds that participants were also concerned about interactions with current medications such as ART, and some were reluctant to add medications to their current regimen, which confirms and extends findings on HCV treatment barriers among HCV mono-infected veterans [6]. Competing stressors, including participants’ desire to treat comorbidities first, or increase stability in other areas, also delayed treatment uptake [6, 30].

On the interpersonal level, peers were an important barrier and facilitator to DAA treatment. Participants reported that information received from peers about interferon-era treatment and observations that some peers who were untreated for HCV had no apparent symptoms were barriers to DAA treatment, similar to other studies [30, 59]. However, many participants said peers who successfully completed DAA treatment motivated them to enter DAA treatment, as one recent study of Irish prisoners also found [32]. Interventions that build on existing social networks for disseminating information have proven successful in optimizing HIV testing, prevention, and treatment [60]. Individuals with HIV/HCV co-infection who have completed DAA treatment, particularly those from marginalized and vulnerable groups, should be encouraged and trained to talk about their experiences with their peers in informal settings and at HIV and HCV support groups. In addition, future research could further examine the influence of peers who have completed treatment on DAA treatment uptake.

On the institutional level, participants described frequent changes in providers, including their HIV healthcare providers, as notable barriers to HCV treatment. Provider turnover can be high in community health centers and other HIV clinic sites. Furthermore, participants frequently access HIV and HCV care in various places and move from one clinic to another (a phenomenon known as “churn”), resulting in changing providers [61]. In contrast, participants emphasized that stable relationships with providers they perceived as trustworthy and concerned for their overall wellbeing facilitated their entry into DAA treatment. These findings are consistent with studies on HCV treatment during the interferon era [62–64] and research on how provider communication skills influence HIV treatment [65]. In addition, some participants noted that being assigned to a new provider with whom they built a strong rapport facilitated their entry into treatment. Notably, some participants who entered treatment after delaying required multiple conversations with their providers before they decided to start DAA treatment. Providers may need to establish relationships over time with patients who delay treatment and allow them to make treatment decisions at their own pace, but persistent encouragement is necessary. Some patients may need a concentrated patient-centered-care approach [66] to facilitate their entry into treatment.

Media messaging is perceived as unrealistic and promotes distrust instead of encouraging DAA treatment. To overcome the influence of media messaging, some participants needed a strong relationship with a provider who gave them comprehensive information on DAA treatment and potential side effects. Providers should be more involved with DAA treatment media messaging to ensure it presents a more detailed and realistic account of DAA treatment and its potential to improve patients’ overall health. For instance, our group’s HCV treatment patient app and website design was designed in collaboration with HIV and HCV treatment providers. Also, different social media approaches that are brief and patient-centered (social-media apps like Tik-Tok or other messaging approaches) should be considered.

On the structural level, the cost of treatment was a barrier for participants, even if they did not bear any financial burden. Because of its expense, some did not feel they deserved treatment or wanted to wait until they felt optimally situated to complete treatment, particularly by improving their health or living situation. Support for medication adherence, particularly the provision of DOT, facilitated DAA treatment. Although none of the participants received DOT specifically for DAA treatment, such an intervention should be considered. Studies have shown that DOT is associated with higher ART adherence and SVR rates [67–69]. Providing patients with DOT for their DAA medication could increase treatment uptake by providing the support some persons with HIV/HCV co-infection may need [31]. The use of telemedicine could also be invoked as a DOT approach.

Many of the themes that shape DAA treatment decisions among persons with HIV/HCV co-infection are similar to those in previous studies with individuals who are HCV mono-infected [37]. However, additional concerns for persons with HIV/HCV co-infection include greater potential for drug-drug interactions with ART which may require adjustment of ART [70, 71]; the role of HIV itself in accelerating liver fibrosis which makes this group a priority population for treatment; and the lower rates of HCV cure in the pre-DAA era for persons with HIV. These clinical issues, combined with the added role of stigma in persons with HIV [72], create additional barriers which need nuanced approaches for promoting treatment. Interventions for increasing DAA treatment uptake for individuals who are mono-infected should be extended to persons with HIV/HCV co-infection, and further studies should examine how interventions may need to be optimized to fit the needs, concerns, and perspectives of persons with HIV/HCV co-infection.

Finally, our analysis shows how the constructivist grounded theory approach can be used to examine barriers and facilitators to DAA treatment for PWH. Using this approach, this study extends the modified social-ecological model into the examination of how multiple themes influence DAA treatment perceptions among PWH.

### Limitations

Our study has several limitations. Interviews relied on self-reported information, thus recall bias may have influenced the accuracy of accounts. Also, since the sample is small and the participating clinics in this study are highly experienced in HIV and HCV treatment, we cannot generalize our qualitative findings to all persons with HIV/HCV co-infection. In addition, since the study included only English-speaking participants receiving care in the U.S., findings may not be generalizable to non-English speaking individuals or persons receiving care in other countries.

## Conclusions

Access to DAA treatment is necessary but not sufficient to meet the World Health Organization’s goals of eliminating HCV. Our study is unique in its focus on patient-perceived barriers and facilitators to HCV treatment among persons with HIV/HCV co-infection who delay or refuse DAA treatment. To increase DAA uptake, interventions should support patients with HCV-treatment adherence, increase DAA treatment knowledge among persons with HIV/HCV co-infection, remove DAA treatment access barriers for persons who actively use substances, and encourage patients who have successfully completed DAA treatment to share their experiences with their peers. For greatest efficacy and lowest cost in time and money, the design of future interventions could benefit from centering the needs and concerns of persons with lived experience.

## Supplementary Information

Below is the link to the electronic supplementary material.

Supplementary file1 (DOCX 22 KB)

## Data Availability

Not applicable.
