# Supplementary material for: Perceptions Towards HCV Treatment with Direct Acting Antivirals (DAAs): A Qualitative Analysis with Persons with HIV/HCV Co-infection Who Delay or Refuse Treatment
Source: AIDS Behav. 2022 Jul 1;27(1):119–33. doi: 10.1007/s10461-022-03749-8 (PMC9663279; doi:10.1007/s10461-022-03749-8)
Supplement: Supplementary file 1 — Supplementary file1 (DOCX 22 KB) [file 10461_2022_3749_MOESM1_ESM.docx]

**Interview Guide**

*Interviews will be a guided conversation. Interviewer will ensure that all areas in the following domains are covered in the interview, either by arising in the conversation or through direct questions from the interviewer. Follow-up questions will be asked to elicit more detailed accounts.*

**Domains are:**

**1. Brief biography and Demographics**

**2. HCV History**

**3. Provider Relationships**

**4. Knowledge and Expectations about HCV**

**5. Perceptions of HCV treatment**

**6. Health and Wellbeing**

**7. Social Support**

**8. Substance Use**

**9. Competing Demands**

**10. Identity**

*I’d like to ask you some questions about your life, and your experience with HCV infection, getting treatment and other types of services that can help you stay healthy, and your thoughts about ways to improve HCV care in your community.*

*Please don’t answer any question that you don’t want to. What I learn from you today will hopefully be used to improve HCV care. Everything we discuss today will be kept confidential. There will be nothing to personally identify you.*

*Do you have any questions before we start?*

*I may take notes during our conversation to help me remember things.*

*Can I turn on the recorder?*

**1. Brief biography, demographics:**

Age, race/ethnicity/gender identity/LQBTQ identity

Parent’s jobs, education

Educational attainment

Work history

Where they’ve lived

Housing history

**2. HCV History**

When did you get diagnosed with HCV?

How did you find out?

Who told you?

How did that go?

How did you feel afterwards?

Did you tell anyone?

How did they react?

Was it different from when you got diagnosed with HIV? How so?

Then what? Did you think about going into treatment? Why/why not?

**3. Provider Relationships**

What providers have you seen in the last month? PCP? Case manager? HIV care provider?

What providers have you seen in the last 6 months?

How did it go last time you were there?

How do you feel about provider x,y,z?

Do you spend enough time with them? Do you talk to them about other concerns? *Probe for provider empathy/ connection, relationships*

**4. Knowledge and Expectations about HCV**

When did you first learn about HCV?

Did you hear anything about HCV treatments back then? Side effects?

What have you heard more recently?

Where else have you learned/ heard about HCV? (Probe for: worried about side effects, bad interactions with HIV meds)

What have you heard of HCV?

Have you talked with any of your providers about HCV?

*Probe for knowledge/concerns about: symptoms, treatment, side effects, time, and efficacy*

How do you feel about what they tell you?

Where else do you get information on HCV?

What have you heard about what it does?

Who did you hear that from? (*Check for: perceptions HCV is benign/unavoidable*)

**5. Perceptions of HCV treatment**

How is your health? *Probe for: overall health, comorbidities, HIV, symptoms, adherence behavior*

Have you had any symptoms from HCV?

How do you feel now about having HCV?

Do you think about being HCV positive much? Do you worry about symptoms? Get tested to see how you are?

Do you feel you’re ready for treatment?

What would it take/what would need to change for you to feel ready for treatment?

Has there been a time in your life where you thought you were ready for treatment?

Has your medical provider suggested treatment for HCV?

What did they say?

What did you think?

Did you trust their opinion?

Do you think they thought you should go on HCV treatment?

Why?

What made you decide to delay treatment?

Are they trained in HIV treatment also?

Is that provider a physician or a nurse or..

Do you think you have better or worse treatment from a nurse/ physician?

Do you think you should ideally be treated now or later?

Was there a time when you were ready for HCV treatment but waited?

What are the reasons you waited?

How do you think HCV treatment should change/could be improved?

*Probe for waiting for new HCV therapies, worried about side effects, bad interactions with HIV medications*)

*Probe for: focused on HIV treatment to exclusion of HCV, want to get HIV under control first.*

*Probe for: do they prefer an HCV provider who knows a lot about HIV too.*

**6. Health and Wellbeing**

How do you feel?

What does being healthy mean to you?

What do you do to keep yourself feeling healthy?

What are your concerns for your health now?

Have you waited for medical treatment in the last 6 months?

What does having a good day mean to you?

How about having a good week?

Are you eating enough lately?

Where do you get food from?

How is your appetite?

Does that affect your medication adherence-

Any side effects from your medications?

Any changes in diet/ digestion from your medications?

Do those bother you?

What do you do to handle the side effects?

Do you think about your other health conditions? If there were a treatment for those, would you go through a 12-week regimen?

Do you ever get depressed- has that influenced your medical care?

**7. Social Support**

Who is in your daily life?

What do you do for them? What do they do for you?

Do they know about HCV? HIV?

Probe for stigma: How did they react? (If they do not know) why haven’t you told them?

Has HCV changed your relationships with them?

What or who do you turn to for support? Probe for: church, friends, family, romantic partner, caseworkers, medical care providers?

Have you known anyone with HCV? Who?

Did they get treated? Did they have any symptoms?

*Probe for: stigma, how it may influence disclosure and support, treatment seeking.*

**8. Substance Use**

Drink alcohol? How often?

How does this affect you making/keeping appointments?

Take opiates- heroin, fentanyl, pills, how often?

How does this affect you making/keeping appointments?

Take cocaine/crack- how often?

How does this affect you making/keeping appointments?

Take speed, meth- how often?

How does this affect you making/keeping appointments?

Use marijuana- how often?

How does this affect you making/keeping appointments?

Do you inject drugs? Have you ever? *Probe for: fear of blood draws because of stigma/stigmata*

Do any of these affect you getting HCV treatment?

Have they affected you getting on or staying on medications?

Have you heard anything about side affects from doing these substances and getting treatment?

Have your medical providers, caseworkers, said anything about these?

Have you thought about stopping or cutting down?

Have you talked to anyone about that?

If you did cut down or stop, do you think you would engage in more or less medical care/ go to medical providers more?

**9. Competing Demands**

Are you currently working or in school?

How does that affect you getting to appointments/ taking meds?

Where are you currently staying at night? *(Probe for housing history, stable housing*)

How does that affect you getting to appointments/ taking medications?

Transportation: How do you get around- do you have a car, walk, take the bus? How long does it take you to get to your appointments? How do you pay for this?

Childcare: (*if indicated children live with them*) Who takes care of your kids while you’re at appointments?

Income: How do you make ends meet?

*Probe for: tax paying job, last employment, gvt assistance, support from friends/ family*

Competing demands: Where does your time go? (*Commitments, conflicts that can get in the way of care, caretaking including children*)

Adherence: How is it for you to make appointments? Do you miss many? Is that an issue for you?

Do you have a daily routine?

**10. Identity**

How do you think living with HIV/ being African American/ being LGBTQ/ using injection drugs/ lacking housing impacts your treatment/relationships with providers? Does it make it harder or easier for you to get treatments and services?

*End interview by thanking participants and asking if there is anything else that they would like to say or that they think we should know.*
